# The Prevalence and Clinical Profile of Hirschsprung’s Disease at a Tertiary Hospital in Bahrain

**DOI:** 10.7759/cureus.12480

**Published:** 2021-01-04

**Authors:** Alaa Ali, Fayza Haider, Saeed Alhindi

**Affiliations:** 1 Pediatric Surgery, Salmaniya Medical Complex, Manama, BHR

**Keywords:** hirschsprung's disease, colonic aganglionosis, hirschsprung-associated enterocolitis, congenital megacolon, transanal pull-through procedure

## Abstract

Background

Hirschsprung’s disease (HD) is a rare but important congenital pediatric disease of the colon, and its incidence varies widely between ethnic groups. Its incidence was first studied in Bahrain in 1980 using hospital-based data. Over a 16-month period, 10 cases were reported, representing a relatively high incidence rate: 1 per 4000 births. Even though the number of live births in Bahrain has increased dramatically over the last four decades (doubling from 10,000 to 20,000 per year), published studies about the incidence of HD are uncommon. In this research, we aimed to determine both the prevalence and the clinical characteristics of HD at a tertiary hospital in Bahrain.

Methods

This retrospective observational cross-sectional study included children diagnosed with HD at a tertiary hospital in Bahrain over the last seven years (2014-2020). Children over 10 years were excluded. Clinical data collected included gestational age, birth weight, gender, associated anomalies, clinical features at presentation, disease management, and complications.

Results

The prevalence of HD in Bahrain was found to be 1.3 per 10,000 live births, according to 18 patients qualified for inclusion in this study. The median age at diagnosis was 18 days; approximately 72% were males, and nearly 94% were Bahraini nationals. Three patients (16.7%) had associated anomalies including cardiac and urogenital malformations. Abdominal distention was the most common clinical presentation (83.3%) followed by constipation (77.8%). Half of the patients (50%) passed meconium within 48 hours of birth. Transanal full-thickness rectal biopsy was the method of diagnosis in most patients (83.3%). Seven patients (38.9%) were diagnosed comparatively late (beyond the neonatal period), at a mean age of 1-2 years. Significant associations between age at diagnosis and clinical presentation, initial management, or surgical intervention were not found. A total of 17 patients (94.4%) underwent the definitive surgery (transanal pull-through procedure). In this surgical group, initial colonic decompression was performed via rectal washout in six patients (35.3%) and via temporary stoma in three patients (17.8%). Laparotomy combined with the definitive surgery was necessary for six patients (35.3%). Most of the patients treated surgically had a short segment disease (70.5%).

Conclusions

The awareness of HD is highly important, especially with more than one-third of cases diagnosed outside the neonatal period and half of them passing meconium within 48 hours of birth. In addition, early detection of HD in the neonatal period would result in a less complicated course by reducing the number of patients requiring a multi-stage surgery and further laparotomies.

## Introduction

Hirschsprung’s disease (HD) is a motor disorder of the gut caused by the failure of neural crest cells to completely migrate during the intestinal development of the fetus. That process begins at week 4 of gestation and ends at week 7 with the arrival of neural crest-derived cells at the distal end of the colon [1]. Failure of this process results in some portion of the aganglionic segment of the colon failing to relax, causing a functional obstruction.

Most cases of HD are diagnosed in the neonatal period, but some present later with persistent and severe constipation [2]. The most common presentation (80%-90%) is failure to pass meconium within 48 hours of birth [2,3]. Other signs of bowel obstruction are also common, such as abdominal distension (76%), bilious vomiting (69%), and feeding intolerance [4].

Worldwide, the incidence of HD approaches 1 to 2 cases per 10,000 live births [4]. Most people live a normal adult life with proper treatment [5]. In a nationwide Japanese survey, HD was estimated to occur in 1 in 5000 live births over 30 years. The male-to-female ratio was 3:1 to 4:1 except when the entire colon was involved, when it was nearly 1:1 [2].

The disease can be associated with neurological, cardiovascular, urologic, or gastrointestinal abnormalities. Down syndrome (trisomy 21) is the most common chromosomal abnormality associated with the disease, occurring in approximately 10% of patients [6]. Early diagnosis is important to avoid complications (e.g., enterocolitis and colonic rupture).

Romani and Khan reported 10 cases of HD in Bahrain over a 16-month period from May 1980 to September 1981 with an incidence of 1 per 4000 births [7]. Since then, there has been a lack of literature on HD in Bahrain despite a rising rate of live births [8]. This study aimed to estimate the prevalence of HD among the infants and children aged less than 10 years at a tertiary hospital in Bahrain during the past seven years, and to describe this group of patients in terms of demographics, birth record data, clinical features, and surgical treatment.

## Materials and methods

In this retrospective observational cross-sectional analysis, we used data from medical records at the Salmaniya Medical Complex in Bahrain. The records of 18 patients diagnosed with HD (defined as the absence of ganglia in the enteric nervous system of the distal bowel) from April 1, 2020, to June 30, 2020, were reviewed. Clinical and operative data were obtained from medical files and the electronic health record system. Inclusion criteria were infants and children aged less than 10 years and admitted to the Salmaniya Medical Complex with HD during the past seven years (2014-2020). A diagnosis of HD was confirmed by histopathological examination of full-thickness rectal biopsies.

Collected data included age at diagnosis, gestational age (preterm vs. full term, defined as at least 37 weeks), maternal age, birth weight, single versus multiple births, nationality, gender, association with Down syndrome or other anomalies, symptoms during the neonatal period, initial management, extent of aganglionosis, type of definitive surgery, and postoperative complications.

The extent of aganglionosis was classified as either short segment, defined as an aganglionic colonic segment limited to the rectosigmoid and upper sigmoid, or long segment, defined as aganglionosis that extends to the splenic flexure or to the transverse colon [6].

The study protocol and waiver of the need for written informed consent were approved by the Secondary Health Care Research Sub-Committee at the Ministry of Health in Bahrain. To maintain patient confidentiality and to ensure privacy, no personally identifying information was collected.

Statistical analysis

IBM SPSS Statistics 23 (IBM Corp., Armonk, NY, USA) was used for data entry and analysis. Categorical variables were described using frequencies and percentages, whereas continuous variables were described using the median, mean, and standard deviation. The Mann-Whitney test was used to find significant differences in medians between two groups, whereas the Kruskal-Wallis test was used to find significant differences in medians between more than two groups. The Fisher exact test was used to find significant associations between two categorical variables. In all statistical analyses, statistical significance was found when the P-value was less than 0.05.

In our study, the prevalence of HD was calculated based on the period prevalence formula per 10,000 live births. The period prevalence is defined as the number of current cases (new and preexisting) over a specified period of time divided by the average or mid-interval population [9].

## Results

The prevalence of HD in Bahrain was found to be 1.3 per 10,000 live births over the past seven years. Baseline characteristics of the 18 patients included in this study are shown in Table 1. The baseline characteristics include the patient’s gestational age, birth weight, gender, nationality, age at diagnosis, and the presence of associated anomalies. Table 1 also describes the maternal age, the mother’s sickle cell status, and the presence of maternal co-morbidities. Notably, children with other anomalies included a female patient with Down syndrome, who additionally had an associated ventricular septal defect as well as bilateral hip dysplasia, a patient with hypospadias and meconium plug syndrome, and the remaining with an undescended testis. In addition, one mother who was a sickle cell carrier was a cystic fibrosis carrier as well. Another mother had hypothyroidism under treatment.

**Table 1:** Clinical characteristics of the study (total = 18) SD, standard deviation. All variables are presented as n (%) for categorical variables or as median and mean ± SD for continuous variables. *Indicates that other anomalies include the patient with Down syndrome, who additionally has an associated ventricular septal defect and bilateral hip dysplasia.

Clinical characteristic		Subcategory	Value
1	Gestational age, n (%)	Term	16 (88.9)
		Preterm	2 (11.1)
2	Birth weight, kg	Median	3.01
		Mean ± SD	3.12 ± 0.64
3	Sex, n (%)	Male	13 (72.2)
		Female	5 (27.8)
4	Nationality, n (%)	Bahraini	17 (94.4)
		Non-Bahraini	1 (5.6)
5	Age at diagnosis, days	Median	18
		Mean ± SD	447 ± 733
6	Down syndrome, n (%)		1 (5.6)
7	Other anomalies, n (%)*		3 (16.7)
8	Maternal age, years	Median	32.0
		Mean ± SD	30.2 ± 4.7
9	Sickle cell status, n (%)	None	15 (83.3)
		Sickle cell trait	2 (11.1)
		Sickle cell disease	1 (5.6)
10	Other maternal co-morbidities, n (%)	None	15 (83.3)
		Hypothyroidism	1 (5.6)
		Cystic fibrosis carrier	1 (5.6)
		Pre-eclampsia	1 (5.6)

Table 2 shows the clinical presentations and diagnoses of HD. The most common presentation was abdominal distention (83.3%) followed by constipation (77.8%). The most common method of diagnosis was a transanal full-thickness rectal biopsy (83.3%).

**Table 2:** Clinical presentation and methods of diagnosis of Hirschsprung’s disease (total = 18) All variables are presented as n (%) for categorical variables.

Clinical presentation		n (%)
1	Failure to pass meconium within first 48 hours	9 (50.0)
2	Bilious vomiting	11 (61.1)
3	Abdominal distention	15 (83.3)
4	Constipation	14 (77.8)
5	Colonic perforation	1 (5.6)
6	Failure to thrive	1 (5.6)
Method of diagnosis		n (%)
1	Full thickness rectal biopsy (transanal)	15 (83.3)
2	Full thickness rectal biopsy (laparotomy)	2 (11.1)
3	Rectal suction biopsy	1 (5.6)

**Figure 1:**
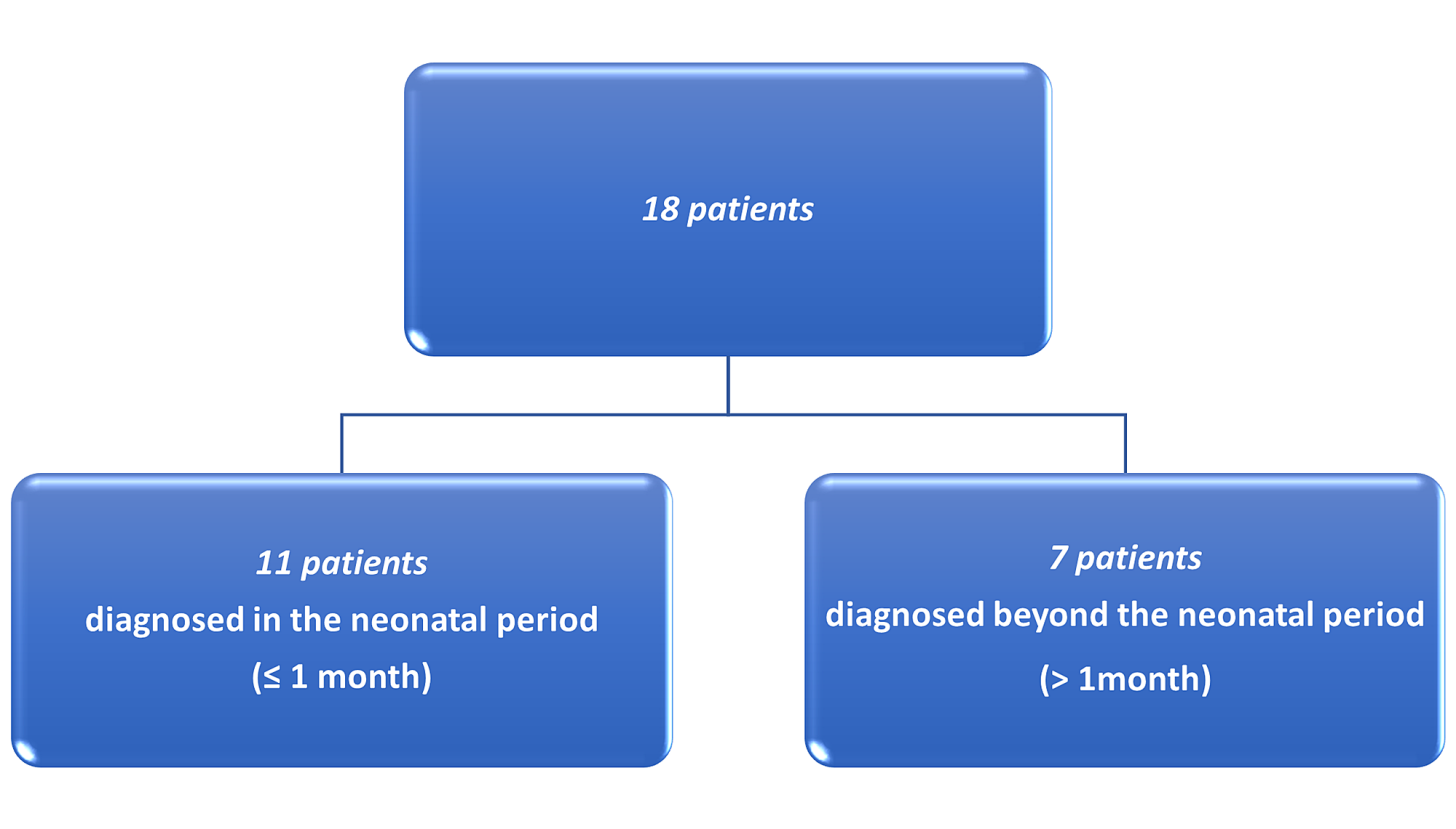
Flow chart showing the numbers of patients diagnosed within and beyond the neonatal period

Among the 18 patients, 11 were diagnosed during the neonatal period (Figure 1), presenting primarily with abdominal distention, bilious vomiting, and constipation. A failure to pass meconium within 48 hours after birth was seen in about half of this subgroup (54.5%). The remaining seven patients (38.9%) were diagnosed comparatively late (beyond the neonatal period), at a mean age of 1-2 years (Figure 1). In this subgroup of patients, the primary symptoms were constipation and abdominal distention; a failure to pass meconium within 48 hours after birth was found in less than half of this group.

**Figure 2:**
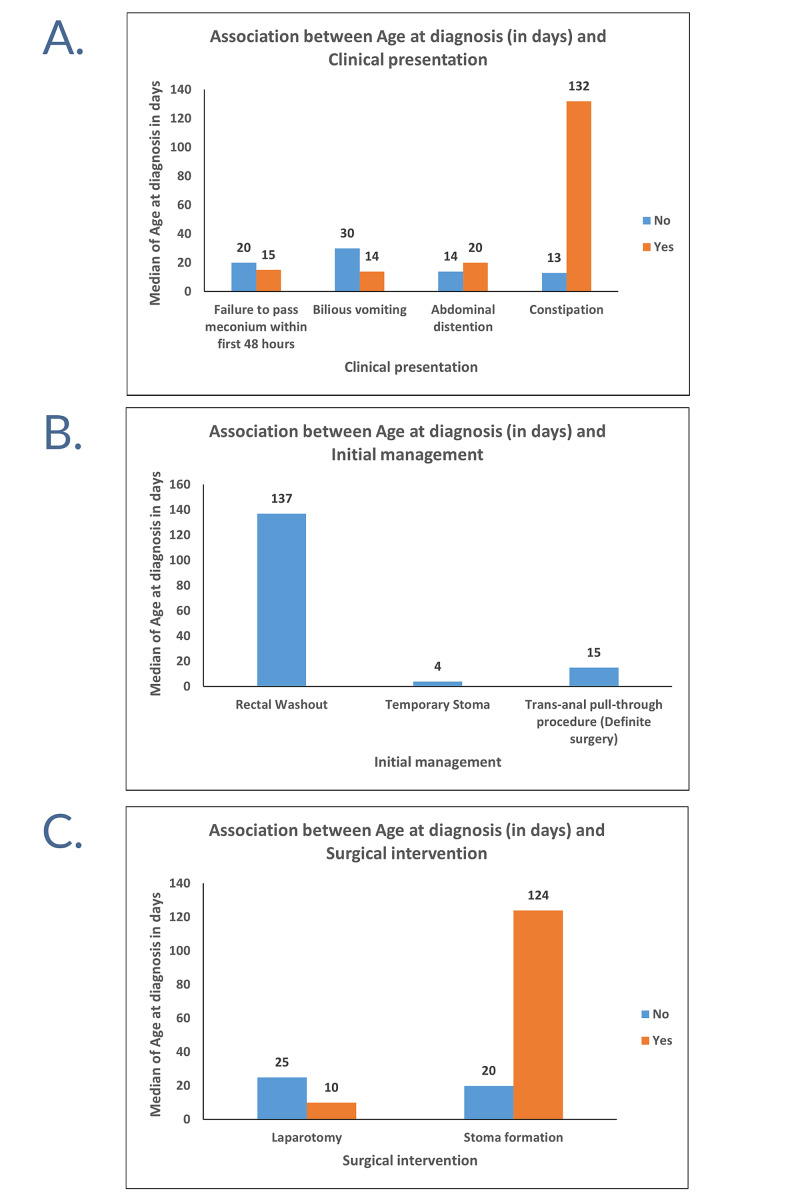
Clustered bar charts of the association between the age at diagnosis (in days) and clinical presentation (A), initial management (B), and surgical intervention (C)

A considerable association could not be found between median age at diagnosis and each of the following: the clinical presentations, the initial management strategy, or surgical intervention (Table 3, Figures 2A-2C).

**Table 3:** Association between age at diagnosis (in days) and clinical presentation, initial management, and surgical intervention SD, standard deviation. All variables are presented as median and mean ± SD, for continuous variables. In all statistical analyses, a P-value of less than 0.05 was considered statistically significant. *Indicates that the P-values were calculated using the Mann-Whitney test. **Indicates that the P-values were calculated using the Kruskal-Wallis test.

Variable		Present (yes/no)	Age at diagnosis, days (median; mean ± SD)	P-value*
A. Clinical presentation				
1	Failure to pass meconium within 48 hours of birth	No	20; 695 ± 934	0.565
		Yes	15; 198 ± 360	
2	Bilious vomiting	No	30; 531 ± 686	0.318
		Yes	14; 393 ± 789	
3	Abdominal distention	No	14; 250 ± 416	0.678
		Yes	20; 486 ± 786	
4	Constipation	No	13; 15 ± 11	0.287
		Yes	132; 570 ± 793	
B. Initial management				
1	Rectal washout	Yes	137; 232 ± 284	0.596**
2	Temporary stoma	Yes	4; 489 ± 841	
3	Transanal pull-through procedure (definitive surgery)	Yes	15; 374 ± 626	
C. Surgical intervention				
1	Laparotomy	No	20; 541 ± 847	0.392
		Yes	127; 348 ± 566	
2	Stoma formation	No	15; 210 ± 372	0.421
		Yes	852; 954 ± 1038	

**Figure 3:**
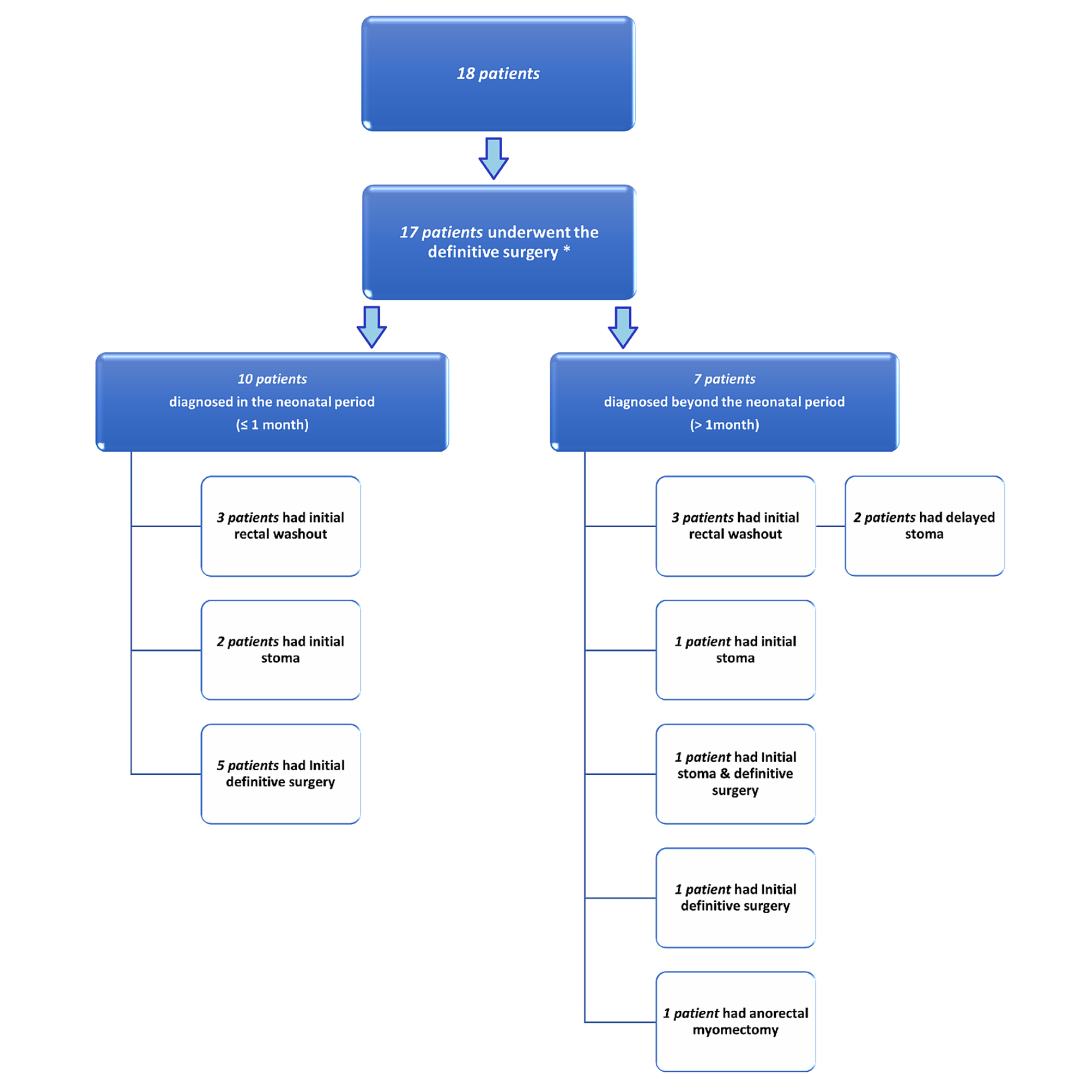
Flow chart showing the management of patients diagnosed within and beyond the neonatal period *Indicates that 1 out of 18 patients refused treatment at our facility and was excluded from the management course.

From the included patients (n = 18), 17 patients underwent the definitive surgery (transanal pull-through procedure), and one patient refused treatment at our facility. This surgery group can be classified into those with neonatal diagnoses (n = 10) and those with late diagnoses (n = 7) (Figure 3). Of the former, two patients (20%) required stoma before the definitive surgery, and three patients (30%) received rectal washouts. Of the latter, four patients (57.1%) required stoma, and three patients (42.9%) received rectal washouts 6 to 12 months prior to the definitive surgery. Comparatively, five patients from the former (50%) received the definitive surgery at diagnosis, as opposed to two patients from the latter (28.6%) (Figure 3). In addition, laparotomy was combined with the main procedure in six patients (35.3%), half from the neonatally diagnosed group (30%) and half from the late diagnosed group (42.9%).

The initial management, surgical intervention, and complications are shown in Table 4. In total, rectal washout was performed as an initial management strategy in six patients (35.3%). A defunctioning stoma was created for bowel decompression in three patients (17.6%), and in two patients, a delayed stoma was required prior to the definitive surgery (Figure 3). The definitive procedure was performed at diagnosis only in seven patients (41.2%). Furthermore, postoperative complications occurred in five patients (29.4%), three developing HD-associated enterocolitis. Other complications were noted such as constipation, fecal soiling, and anal stricture (Table 4).

**Table 4:** Management course of HD HD, Hirschsprung’s disease. All variables are presented as n (%) for categorical variables. *Indicates that 1 out of 18 patients refused treatment at our facility and was excluded from the management course.

Management characteristic		Specific variable	n (%)*
1	Initial management	Rectal washout	6 (35.3)
		Temporary stoma	3 (17.6)
		Transanal pull-through procedure (definitive surgery)	7 (41.2)
		Anorectal myomectomy	1 (5.9)
2	Surgical intervention	Transanal pull-through procedure	17 (100)
		Laparotomy	6 (35.3)
		Stoma formation	6 (35.3)
3	Extent of aganglionosis	Short segment	12 (70.5)
		Long segment	5 (29.4)
4	Surgical complication	No complication	12 (70.6)
		HD-associated enterocolitis	3 (17.6)
		Constipation	1 (5.9)
		Anal stricture	1 (5.9)

Most of the patients treated surgically had a short segment disease (70.5%), and the remainder had long aganglionic segments (Table 4). The male predominance was more prominent in short segment disease with a male-to-female ratio of 5:1, compared to a ratio of 1.5:1 for long segment disease. Nevertheless, the association between the patient’s gender and the extent of aganglionosis did not reach statistical significance (Table 5). We also noted that HD-associated enterocolitis occurred more frequently in children with long segment disease (66.7%), as opposed to those with short segment disease (21.4%). This difference was not statistically significant (Table 5).

**Table 5:** The association between the extent of aganglionosis with the patient’s gender, and the presence of HD-associated enterocolitis HD, Hirschsprung’s disease. All variables are presented as n (%) for categorical variables. In all statistical analyses, a P-value of less than 0.05 was considered statistically significant. *Indicates that 1 out of 18 patients refused treatment at our facility and was excluded from the management course. **Indicates that the P-values were calculated using the Fisher’s exact test.

Variable		Extent of aganglionosis		P-value^**^
		Short segment	Long segment	
		n (%)	n (%)*	
Gender	Male	10 (76.9)	3 (23.1)	0.538
	Female	2 (50)	2 (50)	
HD-associated enterocolitis	Yes	1 (33.3)	2 (66.7)	0.191
	No	11 (78.6)	3 (21.4)	

## Discussion

In this study, we establish an entry point to determine the epidemiological features of HD in Bahrain based on the currently available data. Case ascertainment was optimally maintained by precise information gathering from medical records. The prevalence of HD in Bahrain, 1.3 per 10,000 live births, is consistent with the worldwide incidence, 1 to 2 per 10,000 live births [5]. In addition, we found male predominance among our patients with a male-to-female ratio of 2.6:1, which is in line with the international reported ratio of 2.9:1 to 4.5:1 [2,10].

HD usually presents in neonates with an intestinal obstruction that is accompanied by abdominal distension, bilious vomiting, and feeding intolerance during the first few days of life; 45%-90% of patients present with a failure to pass meconium within 48 hours of birth [11,12]. Less frequently, patients with HD present later in childhood, typically complaining of chronic constipation that is often refractory to laxatives. A failure to thrive may be seen as well [13]. Another mode of presentation is enterocolitis, a potentially life-threatening condition with symptoms ranging from mild disease indistinguishable from gastroenteritis to a sepsis-like picture that may be fatal [13]. In our study, we noted that HD can have a variable clinical presentation. For instance, nine patients (50%) passed meconium within 48 hours of birth. In addition, the ‘classic triad’ of delayed passing of meconium, abdominal distension, and bilious vomiting was noted only in five patients (27.8%).

HD has a complex pathophysiology that is affected by numerous aspects including genetic and maternal factors [14-16]. Heritability in HD has been identified in more than 80% of cases; associations with sequence variants in genes related to the enteric nervous system as well as monogenic and chromosomal syndromes have been found [14]. In individuals with HD, the reported overall incidence of Down syndrome was 7.32%, and 50% had associated congenital heart disease [15]. Our study group included one patient with Down syndrome, and in this case, an associated ventricular septal defect and bilateral hip dysplasia were present. This individual was found to have a long segment disease. This is in line with other studies that reported that HD with coexisting Down syndrome had significantly higher rates of long segment disease [15].

As for the maternal risk factors, maternal obesity and parity were found to be associated with a higher risk for HD, and affected children were born at lower gestational ages than controls [16]. However, advanced maternal age was not associated with an increase in the rate of HD, despite being a risk factor for genetic diseases such as Down syndrome [16]. The maternal age across our entire study group was less than 36 years (the mean maternal age at birth was 30 ± 4 years). In addition, we noted that maternal comorbidities including sickle cell disease and hypothyroidism were present in a few patients. Nevertheless, further studies with a larger sample size are warranted to verify an association between such comorbidities and HD.

The diagnosis of HD is confirmed via an examination of rectal biopsy specimens [17]. Although tissue specimens taken by suction rectal biopsies are lesser than those by full-thickness rectal biopsies, they provide similarly reliable diagnoses [17]. We used full-thickness rectal biopsies in 17 patients, and a suction rectal biopsy was used to diagnose the remainder.

Early diagnosis in the neonatal period is highly important to avoid complicated presentations such as HD with enterocolitis and colonic perforation [18]. In our study, the mean age at diagnosis was 12 months. We found that seven patients were diagnosed beyond the neonatal period for various reasons. Misinterpretations of the rectal biopsies played a role in late diagnosis [19]. This was reported in two patients; in one of them, an initial rectal biopsy showed ganglion cells, and for another, that biopsy was inconclusive. In addition, a delayed HD diagnosis could be the result of an associated meconium plug syndrome, which was the case in one of our patients who was misdiagnosed with this syndrome in the neonatal period instead of HD. Another reason for late diagnosis could be explained by patients presenting atypically with chronic constipation or abdominal distention between the ages of two and four years [11]. Furthermore, we noted that delayed diagnosis was associated with a complicated course. For instance, an infant developed a closed-loop obstruction with a partial right colonic volvulus at the age of 11 months (three months after diagnosis) and underwent laparotomy and colostomy. Another patient underwent anorectal myomectomy once diagnosed (at three years of age), and, at nine years of age, he developed severe abdominal distention due to constipation that did not respond to a rectal washout and fecal disimpaction. He was eventually managed with a laparotomy and colostomy followed by the definitive surgery at 10 years of age.

The treatment of HD includes numerous types of surgical operations [20,21]. The transanal pull-through procedure is the main definitive surgery that is used in HD treatment. It can be performed as a one-stage operation or a multi-stage surgery. The one-stage transanal endorectal pull-through procedure, performed without opening the abdomen, has recently been used with excellent outcomes [20]. This procedure is optimally done within the neonatal period and has many advantages. It has lower rates of post-operative pain, less use of analgesia, shorter hospital stays, lower risk of adhesive intestinal obstruction, and better cosmetic outcomes without visible scars [21]. Achieving earlier diagnosis of HD can allow surgeons to intervene surgically within the neonatal period using the one-stage transanal endorectal pull-through procedure, avoiding any additional complications. In contrast, multi-stage surgeries, such as initial colostomy and laparotomy, carry a higher rate of postoperative complications [20,21]. In our study group, the indications for multi-stage surgery included fecal impaction requiring a diverting colostomy or dismantling colostomy in two patients. Another patient underwent closure of a stoma. Further laparotomies were also needed to mobilize the colon in two patients.

## Conclusions

The prevalence of HD in Bahrain is 1.3 per 10,000 live births, and it corresponds to the worldwide incidence. Awareness of HD is crucial and of vital importance due to its variable clinical presentation with at least one-third of patients diagnosed beyond the neonatal period and half of them passing meconium within 48 hours of birth.

Early detection and definitive diagnosis within the neonatal period can reduce the complication rate, and lead to a better disease outcome. It also allows prompt surgical intervention using the one-stage transanal endorectal pull-through procedure, thus avoiding the need for multi-stage surgeries and further laparotomies that carry a higher rate of postoperative morbidity.
